# Supplementary material for: Flow rate of transport network controls uniform metabolite supply to tissue
Source: J R Soc Interface. 2018 May 2;15(142):20180075. doi: 10.1098/rsif.2018.0075 (PMC6000175; doi:10.1098/rsif.2018.0075)
Supplement: Supplementary Material [file rsif20180075supp1.pdf]

S1. APPROXIMATION FOR ABSTRACTION OF PLANT LEAF XYLEM FLOW

For the theoretical approach employed here, the sap flow in the plant leaf xylem network is not considered in its full complexity, but approximations are made abstracting the characteristics of the plant leaf xylem flow network to allow for an analytic treatment. We are interested in the constraints active transport in a network puts on the metabolite supply in a tissue. For this reason, we focus on the transport in the intravascular pathways. As stated in section S3 A, for the calculation of the absorption along a vessel, the flow velocity in individual tubes is calculated using Kirchhoff's circuit laws. The calculation of the flows using Kirchoff's circuit laws, however, demands idealizations of the xylem vessel network regarding the distribution of the outflow sites and the vessel geometry. Focusing on the the outflow, fluid is evaporating through small pores, called stomata, that are distributed evenly over the surface of the leaf. As stomata are not connected to the xylem network, the fluid is leaving the xylem vessel and flows through the extravascular tissue to the stomata sites. Considering a uniform distribution of stomata and the same outflow of fluid at every stomata, the amount of fluid leaving each tube can be estimated. However, Kirchoff's circuit law demands that outflow is a property of nodes, while no fluid loss is allowed along tubes of a network. Integrating the total stomata outflow and redistributing it back to the nodes, such that every node has the same outflow of fluid, results in flow profiles consistent with the estimate of a steady outflow of fluid along each tube. The redistribution of the outflow sites is well accepted in the literature [1, 2]. As we consider steady state solutions, we assume a balance between total inflow of fluid in the network and outflow out of the network. For short time scales this balance is not expected to hold in real plants given the storage capacities of the tissue. Yet, here we are interested in long time scales warranting the balance between in- and outflow. Focusing on the vessel geometry, application of Kirchoff's circuit laws requires an estimate of the hydraulic resistance along each vessel. To this end, xylem vessels are estimated as circular straight tube.

For given inflow and outflow at every node, the pressures at every node and thus the flow between nodes are fully defined, as Kirchhoff's circuit law is applied. Note, that setting both the pressure and the flow values at the nodes at the same time overdetermines the system, as flow values and pressure values are determining each other consistently. We chose flow values as input to model stomata conductance and calculated the pressures. The calculated pressures result in pressure drops between tubes connecting nodes, which then results in fluid flow between these nodes.

The flow resulting from this calculation is in agreement with an observed linear dependence of the total pressure drop with the total inflow of solute [3], see Fig. S1(a). Fig. S1(a) shows the pressure difference between the first inflow node and the last outflow node on the bottom side of the network for three different inflow rates using the networks shown in Fig. 2. A regression through the origin results in a slope of $\approx 0.015\,\mathrm{s\,Pa\,\mu m^{-3}}$. In addition our model agrees with observations [3] of a steady decrease of the flow velocity along the network length as the distance to the inflow site increases, see Fig. S1(b). The profile is taken along the center vertical axis of the rectangular network excerpt of the network with optimal inflow as shown in Fig.2.

S2. ARTIFACTS IN SUPPLY PATTERN DUE TO SYMMETRIES IN NETWORK EXCERPT

For the rectangular network excerpt, we consider a tessellation with small triangles resulting in a highly interconnected network. To avoid artifacts in supply patterns originating from a high intrinsic symmetry, all node positions were slightly randomized. Examples of artifacts in supply patterns are visualized in Fig. S2. Fig. S2 (a) shows a non-randomized network that otherwise has the same network parameters and influx rate as the network in Fig. 2 (b). In contrast to the randomized network, the non-randomized network shows a clearly symmetric absorption pattern, as the absorption near the axis is decreased and the absorption near the left and right margin of the network is increased. Comparing the flow profiles of the two networks along a horizontal row, as indicated by a blue line in Fig. S2 (a), shows a high regularity for the non-randomized network, see Fig. S2 (b). The fluctuations of the flow velocity are stronger in the randomized network, see Fig. S2 (c). While for the non-randomized network the flow in tubes in the direction of the boundaries of the network is strictly faster than in tubes that lead flow toward the center of the network, the same tendency is still observable in the randomized network, though here not strictly true anymore. Faster flow to the margins of the network, results in a stronger displacement of metabolites to the margins. This is observable for both Fig. 2 (b) and much more amplified Fig. S2 (a). This effect results from the assumption that no further in and outflow of fluid is considered on the side of the network excerpt at the margins.

S3. NUMERICAL METHODS

A. Calculation of absorption profiles in a network

For the calculation of absorption profiles the absorption along each tube of the network was calculated individually. The gist of the calculation is to apply Eq. 6 on each tube of the network. For a given network topology, we consider all variables of Eq. 6 as known, with exception of the average flow velocity and the inflow of metabolites in a tube. The calculation of the absorption profile is hence following two steps:

1. First using Kirchhoff's circuit laws, we compute the velocity profile in the network. Besides the flow velocity in each tube, the velocity profile also states for each node all tubes that start or end at that node.

2. The absorption profile is calculated by iterating through the nodes of the network. In each iteration step, it is determined whether the absorption is calculated in all tubes ending at a node. If this is the case, the absorption in all tubes starting from this node is calculated. Otherwise the node is ignored and the next node is analyzed until absorption in all nodes has been determined. The iterations starts with the tubes starting at an inflow node.

We focus on steady state solutions only. As a consequence, all metabolites flowing into an node are redistributed proportional to diffusion and flux in the tubes starting from this node. The surface integrals of the flux over the cross-sectional area A of each tube at a node point k have to add up to zero: $\sum_{i\in\text{tube}}^{\text{ends in } k} J_i A_i = \sum_{i\in\text{tube}}^{\text{starts in } k} J_i A_i$. In each individual tube the flux of metabolites is proportional to the metabolite concentration, Eq. (5), $J = C(U + \kappa\beta/\ell)$. This determines the influx of metabolites in tubes branching from a node with $J_0 = \tilde{C}_n(U + \kappa\beta/\ell)$. We understand $\tilde{C}_n$ as a node concentration with $\tilde{C}_n = \sum_i^{\text{inflow}} J_i A_i / \sum_k^{\text{outflow}} (U_k + \kappa\beta/\ell_k) A_k$, summing over all inflowing and outflowing tubes, respectively. The metabolite outflux at the end of a tube is given by the difference of metabolite influx and total absorption along the tube. Finally, at the lower end of the considered network excerpt, opposite the inflow nodes, remaining metabolites are flowing out of the network. Since the outflowing metabolites would lead to an accumulation of metabolites, we state the amount of metabolites not absorbed for every considered network excerpt.

B. Optimization of the network architecture

In contrast to the absorption profiles, the optimized network topology is simulated by iteratively optimizing the network topology for uniform absorption. As the network couples absorption rates with the network's flow profile, no closed analytic formulation can be derived for the optimization of the network architecture.

To numerically optimize for a uniform supply pattern, we minimize the differences in absorption $\phi_i - \phi_j$ among all tubes. To penalize especially large differences in absorption, we sum the exponential of differences in absorption and define a score function by

$$\mathcal{H}\{\phi_n\} = \sum_{i,j}^{N} \exp((\phi_i - \phi_j)^2/\alpha), \tag{S1}$$

where $\alpha = \langle\phi\rangle^2$ is a normalization factor. For the sake of comparability of different network architectures and supply patterns, we also penalize metabolite outflux at the end of the network by an additional factor. The difference in absorption $\mathcal{H}$ is multiplied by $(1 + f(J_{\text{out,tot}}/J_{\text{in,tot}}))$, where $J_{\text{out,tot}}/J_{\text{in,tot}}$ is the percentage of outflowing metabolite. A functional form of $f(x) = \exp(1/x)$ is chosen to penalize higher outflux stronger than lower outflux. This score is used to create a potential landscape with a dimensionality proportional to the number of tubes in the considered network. The aim is to find the global minimum of the potential landscape. However, this is not feasible due to the high dimensionality of the landscape. To approach this problem, we used a stochastic Metropolis-Hastings sampler combined with simulated annealing in order to find local minima of the potential landscape. Optimized network topologies were determined for different inflow rates. The usage of Monte-Carlo methods allows a reduction of the computational time. This makes estimates of optimized topologies feasible.

The gist of the Monte Carlo sampler is to randomly choose an alternated network topology and calculate the similarity score. By this, the sample space is probed for the minimal value. For systematic sampling that allows an ergodic coverage of the sample space while reducing the computation time, a Metropolis-Hasting algorithm combined with simulated annealing is used. The idea of the algorithm is to chose a new sample close to the last accepted sample point. If the new sample point has a smaller score according to Eq. S1, the new sample point is accepted. If the new sample point has a bigger score, the sample point is accepted proportional to an exponential distribution with $p \propto \exp(-\beta_T(\mathcal{H}\{\phi_n\}_{\text{new}} - \mathcal{H}\{\phi_n\}_{\text{old}}))$. Here the factor β_T is a parameter that determines how often upward fluctuations appear. Upward fluctuations are needed to allow the algorithm to cross potential barriers. This prevents the algorithm from being trapped in local minima and guarantees an ergodic sampling of the whole space.

To tackle the potential hierarchy in tube patterns, we only allow the change of a random cluster of adjoining tubes. The usage of clustered changes is also suited to find inhomogeneous and hierarchical patterns, which we did not find here in the end. Changing the number of tubes N_c in a cluster changes the proximity of the new sample point to the last accepted sample point. For all chosen tubes the start and end node are slightly changed by addition or subtraction of a small value drawn from a normal distribution with a variance of a one-twentieth of the initial average tube length ℓ. The radius is dilated or constricted by addition or subtraction of a small value Δ for all chosen tubes. The value of the change is a fraction of the current radius of the tube, here denoted as *fraction of change.* The fraction of change is distributed uniformly where F_c denotes the maximal fraction of change. Whether a tube is dilated or constricted is chosen randomly.

To achieve faster convergence to minima, a heuristic argument can be used to bias the choice of tube dila-

tion or constriction. As the radius of a tube is reduced, the wall area is reduced and hence the absorption is decreased. Note, that this does not hold for all tubes as the problem is highly coupled but presents a good rule of thumb. Implementing a biased choice for the dilation or constriction, the acceptance probability has to be changed for the algorithm to remain ergodic.

If $\phi_i > \bar{\phi}$, the probability to expand a tube i by Δ is $p_{i+} = 1/3$, whereas the probability to dilate the tube by Δ is $p_{i-} = 2/3$ and vice versa for $\phi_i < \bar{\phi}$. Here, $\bar{\phi}$ is the mean absorption over all tubes given by the last accepted topology. Using this procedure to chose the next state $x_{(i+1)c}$, we have to estimate the fraction $p(x_{(i+1)c}|x_i)/p(x_i|x_{(i+1)c})$. This fraction is dependent on the sample $(\phi_j)_N^i$ and on the sample $(\phi_j)_N^{i+1}$. Since the choice of dilating or expanding a tube k is solely dependent on ϕ_k^i, we can factorize

$$p(x_i|x_{(i+1)c}) = \prod_k^{\text{tubes}} p(x_{i_k}|x_{(i+1)c_k}) = \prod_k^{\text{tubes}} p_k(\phi_k^i).$$

For tubes which change the regime from *below average* to *above average* the fractions $p_k(\phi_k^i)/p_k(\phi_k^{i+1}) = 1$ cancel out. The fraction is hence given by the number of tubes which stayed *above average* or which stayed *below average* and made the favored step. Given the number of tubes which stayed in their regime and the number of tubes which made the unfavored step, we can express the fraction by

$$M = \frac{p(x_{(i+1)c}|x_i)}{p(x_i|x_{(i+1)c})} = 2^{\#\text{tubes, remained}-2\cdot\#\text{unfav. changes}}.$$

The measured change has to be multiplied to the acceptance probability, such that

$$p \propto M \cdot \exp(-\beta_T(\mathcal{H}\{\phi_n\}_{\text{new}} - \mathcal{H}\{\phi_n\}_{\text{old}})).$$

To identify optimal network architectures, we iteratively reduce the fluctuations within the Metropolis-Hasting algorithm by employing simulated annealing. Our simulated annealing algorithm has five phase. In each new phase the proximity of the new sample points is increased, as F_c and N_c are reduced. Also the frequency of upward fluctuations is decreased as β_T is increased. The first phases are used to allow for strong fluctuations to overcome large barriers in the potential landscape. In the later phases the best minimum is finer and finer approximated, see Fig. S3. The idea of simulated annealing is inspired by the physical picture of crystallization, where the crystal is partly melted to improve the homogeneity of the crystal structure.

Beside minimizing the outflux of solute, we set the constraint of a constant surface area of the network. This constraint is equivalent with conservation of the total material used to build the network. We enforce this constraint by estimating the total difference in the radial R and length ℓ distribution and in a second step dilate or expand all tubes by the same amount such that the total difference equals zero.

We allow in our simulation for cutting of tubes and thus for modification of our initial network topology. Hence, we define a cutoff parameter R_{cut}. If any radius is smaller $R_i < R_{\text{cut}}$, this tube is regarded as cut and $R_i = 0$. Cutting of tubes that would result in unconnected parts of the network with the remaining network was prohibited. The initial state of the network is a mesh, representing a tesselation of space, with randomly chosen radii distribution.

To achieve convergence to a low minimum the algorithm parameters β_T, N_c, and F_c have to be estimated for each annealing phase of the algorithm. Here strong fluctuations should be observable in the first phase, while almost no fluctuations to higher similarity scores should be observable in the last phase. Note, that for changes in the dimension of the to be optimized topology, the algorithm parameters have to be reevaluated and adjusted. For our optimization, an initial value of $\beta_T = 25$ was chosen. This value was increased in each phase as the value of the previous was multiplied by a factor of 5. A value of $N_c = 50$ was chosen and not changed for different phases. Fraction of change was initially chosen as $F_c = 0.1$ decreased by a multiplication with 0.75 for each phase. A total number of 60000 samples was considered for each inflow rate. All of the 5 annealing phases were all of the same length.

S4. VERIFICATION OF THE APPROXIMATION IN ANALYTICAL CALCULATION

For the verification of the three analytical approximations made in section *Metabolite absorption across a fluid filled tube*, we identify three dimensionless parameters, which all have to be much smaller than one for the approximations to hold:

$$R \cdot \gamma \ll 1, \tag{S2}$$

$$\frac{R^2\langle U\rangle}{\kappa\ell} \ll 1, \tag{S3}$$

$$R/\ell \ll 1. \tag{S4}$$

As these parameters have to hold for all tubes, we determine the maximal value of these three parameters for each network considered in the paper. If the maximal values are much smaller than one, then all tubes in the network will fulfill the conditions proposed in the approximations. The values are evaluated for Fig. 2 and Fig. 4 in the paper. The values are listed in Tab. S1. The approximations are also tested for an altered parameter range, see Fig. S4 and Fig. S5. All networks agree with the approximations made.

TABLE S1. Verification of approximations

	Low inflow	Medium inflow	High inflow
Uniform radius networks Fig. 2			
$\max(R\gamma)$	3×10^{-5}	3×10^{-5}	3×10^{-5}
$\max\left(\frac{R^2\langle U\rangle}{\kappa\ell}\right)$	1.2×10^{-3}	5.0×10^{-3}	9.1×10^{-3}
$\max(R\ell)$	4.3×10^{-2}	4.9×10^{-2}	4.4×10^{-2}
Optimized radius networks Fig. 4			
$\max(R\gamma)$	1.6×10^{-4}	1.4×10^{-4}	1.1×10^{-4}
$\max\left(\frac{R^2\langle U\rangle}{\kappa\ell}\right)$	6.5×10^{-3}	9.6×10^{-3}	17.6×10^{-3}
$\max(R\ell)$	0.176	0.162	0.107
Uniform radius networks (altered parameter range) Fig. S4			
$\max(R\gamma)$	2.1×10^{-4}	2.1×10^{-4}	2.1×10^{-4}
$\max\left(\frac{R^2\langle U\rangle}{\kappa\ell}\right)$	0.8×10^{-2}	2.6×10^{-2}	4.1×10^{-2}
$\max(R\ell)$	2.5×10^{-2}	2.4×10^{-2}	2.3×10^{-2}
Optimized radius networks (altered parameter range) Fig. S5			
$\max(R\gamma)$	1.3×10^{-3}	7.0×10^{-4}	8.9×10^{-4}
$\max\left(\frac{R^2\langle U\rangle}{\kappa\ell}\right)$	3.1×10^{-2}	4.5×10^{-2}	0.23
$\max(R\ell)$	0.15	0.065	0.19

S5. SUPPLY PATTERNS QUALITATIVELY INDEPENDENT OF PARAMETER CHOICE

To show that our theoretical framework holds for a wide range of parameters, we exemplarily changed the parameters used in Fig. S4 by one order of magnitude from $\ell = 0.1\,\mathrm{mm}$ to $\ell = 1.8\,\mathrm{mm}$ and from $R = 3\,\mathrm{\mu m}$ to $R = 30\,\mathrm{\mu m}$. The order of magnitude of the total inflow rate is chosen to yield velocities observable in lower order xylem vessels $\langle U\rangle_r \approx 1\,\mathrm{\mu m\,s^{-1}}$. We vary the fluid inflow rate from $Q_{\mathrm{in}} = 1 \times 10^{-4}\,\mathrm{mm^3\,s^{-1}}$ to $Q_{\mathrm{in}} = 5 \times 10^{-4}\,\mathrm{mm^3\,s^{-1}}$. For molecular diffusivity, we keep a value of $\kappa = 1 \times 10^{-10}\,\mathrm{m^2\,s^{-1}}$ as we consider small molecules. The network size was not altered with $N \approx 1000$ tubes considered for the network. For the absorption parameter, we consider $\gamma = 7\,\mathrm{m^{-1}}$. All approximations made in the section *Metabolite absorption across a fluid filled tube* hold also for this parameter choice as demonstrated in Tab. S1. Comparison of Fig. S4 and Fig. S5 with Fig. 2 and Fig. 4 show qualitative agreement for the considered range of the parameter choice. This is in agreement with the scaling prediction of Fig. 3.

[1] F. Corson. Fluctuations and redundancy in optimal transport networks. *Phys. Rev. Lett.*, 104(4):048703, Jan. 2010.

[2] E. Katifori, G. J. Szöllősi, and M. O. Magnasco. Damage and fluctuations induce loops in optimal transport networks. *Phys. Rev. Lett.*, 104:048704, Jan 2010.

[3] M. A. Zwieniecki, P. J. Melcher, C. K. Boyce, L. Sack, and N. M. Holbrook. Hydraulic architecture of leaf venation in Laurus nobilis L. *Plant, Cell & Environment*, 25(11):1445–1450, Nov. 2002.

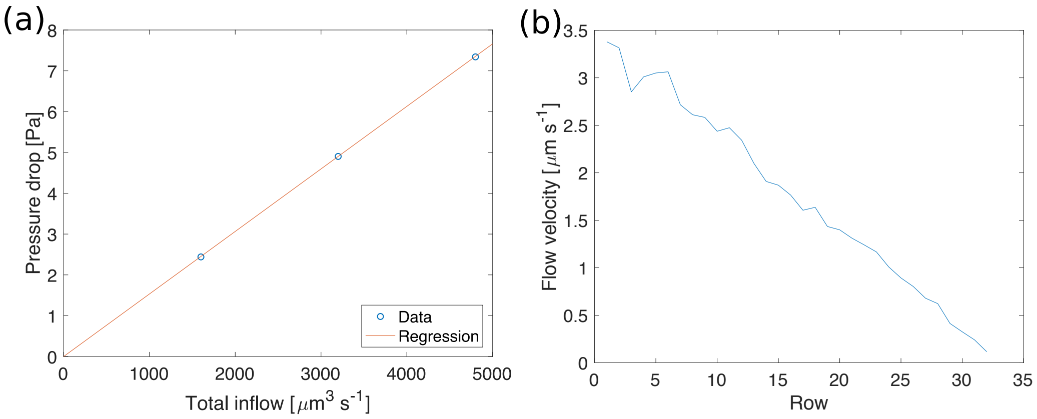

FIG. S1. (a) Linear dependence of the pressure drop over the network with inflow rate. Pressure drop is measured as the difference in pressure between the first inflow node and the last outflow node on the bottom of the network, see Fig. 2. The regression through the origin has a slope of $0.015\,\mathrm{s\,Pa\,\mu m^{-3}}$. (b) Flow profile along the center vertical axis of Fig. 2(b). With increasing distance from the inflow node the flow velocity decreases.

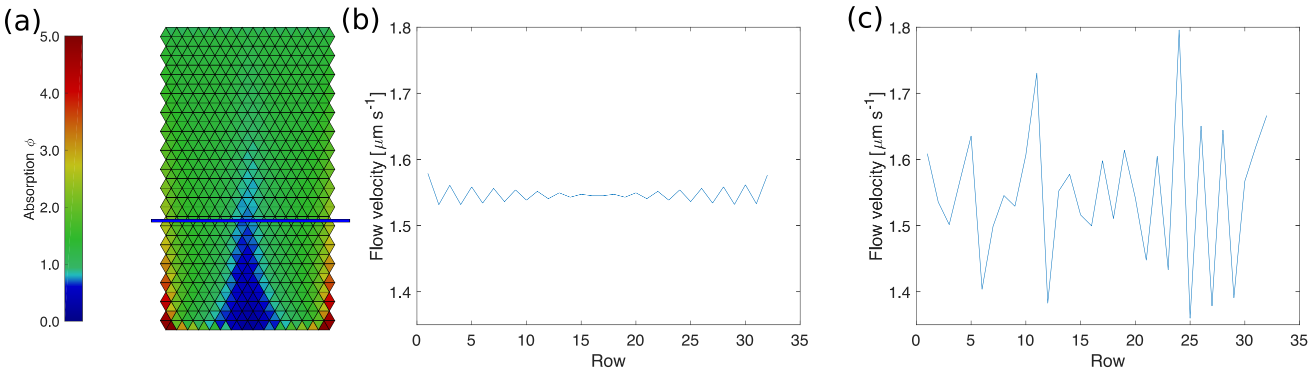

FIG. S2. (a) Absorption pattern in a non-randomized triangulated network showing a clear pattern that is due to the high symmetry of the network. Randomization of network node positions removes pattern, therefore the pattern is identified as artefact due to high degree of symmetry. Flow velocity profile along a horizontal axis for a non-randomized (b) and randomized network (c) as indicated by blue horizontal line in (a) and correspondingly Fig. 2b. The parameter settings are the same as for Fig. 2 for optimal inflow.

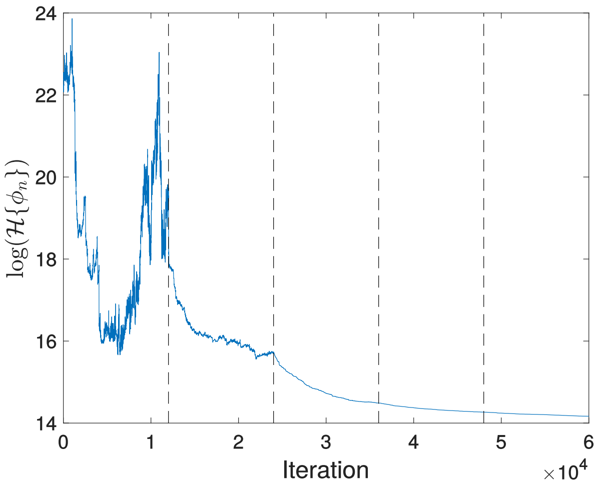

FIG. S3. Exemplary plot of the score function development over time. Every data point shows the score of the last accepted sample point at a time point. We implemented simulated annealing with five phases, where initial phases allow for large fluctuations necessary to cross potential barriers, followed by later phase with only small fluctuations resulting in a finer estimation of the found minimum. Parameters as in the slow inflow example in Fig. 4. Vertical dotted lines indicate different phases of the simulated annealing.

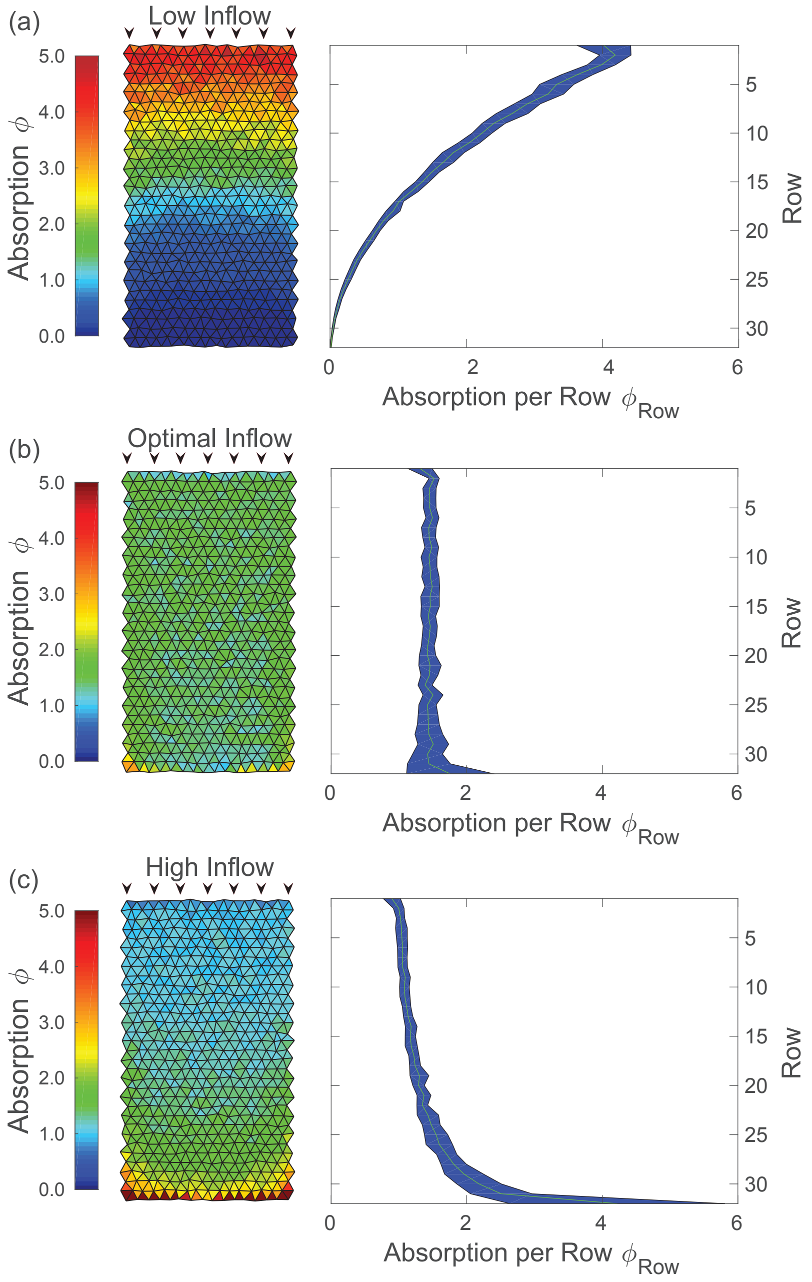

FIG. S4. Supply patterns are controlled by fluid inflow rates. Supply pattern of a rectangular tissue section pervaded by a transport network for increasing fluid inflow rate ranging from (a) $Q_{\text{in}} = 1 \times 10^{-4}\,\text{mm}^3\,\text{s}^{-1}$, via (b) $Q_{\text{in}} = 3.3 \times 10^{-4}\,\text{mm}^3\,\text{s}^{-1}$, to (c) $Q_{\text{in}} = 5 \times 10^{-4}\,\text{mm}^3\,\text{s}^{-1}$. The transport network is build of tubes of equal radius and roughly equal length triangulating the tissue section under consideration. Left column: Supply pattern in every triangulated tissue section given by the average metabolite absorption along neighboring tubes. The absorption is normalized with the inverse of the total influx J_{tot}^{-1} and the total number of tubes $\mathcal{N}$. Right column: Standard deviation and mean absorption per row counting downward from the inflow nodes at the top of the network. At low inflow rate (a) metabolites are absorbed close to inflow and are not transported through the network while for high inflow rate (c) metabolites get flushed through the network for being absorbed mainly at the end. The variance in absorption across all tubes is 0.9 for low inflow rate and 0.33 for high inflow rate. In between these two cases an optimal inflow rate with the lowest variance exists (b) that yields uniform supply and a overall variance of only 0.07. Metabolites are absorbed across tube walls into the tissue, few remaining metabolites are flowing out at the bottom end amounting to 0.03%, 0.8%, and 3.15% for (a), (b), and (c), respectively.

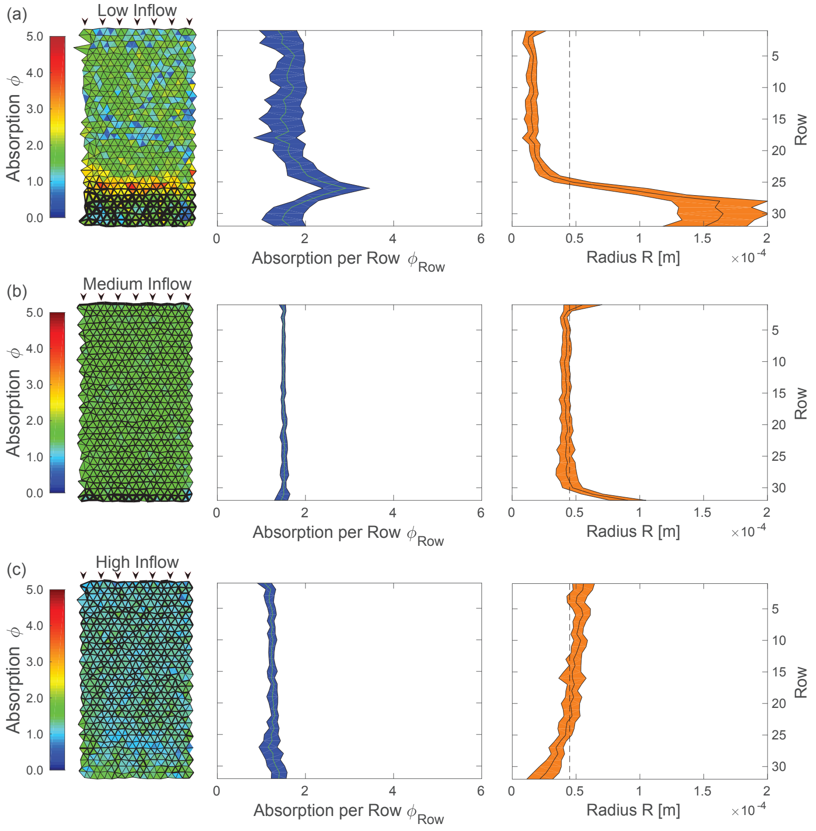

FIG. S5. Optimized network architectures for uniform metabolite supply patterns. Supply pattern for the same low (a), optimal (b) and high (c) inflow rate as in Fig. S4 but optimized network architecture. Left column: Supply pattern in every triangulated tissue section given by the average metabolite absorption along neighboring tubes, see also FIG S4. Thickness of tubes represents the tube radius. Middle column: Standard deviation and mean absorption per row counting downward from the inflow nodes at the top of the network. Right column: Standard deviation and mean radius per row. Dashed line marks average tube radius. (a) For low inflow rate tubes contract near inflow nodes, speeding up flows there and thus propagating metabolites further down the network. Tubes dilate toward the network end further increasing absorption there. Variance in absorption is reduced by almost an order of magnitude down to 0.12. (c) For high inflow rate tubes dilated close to the inflow nodes, slowing down flow there and thus increasing absorption. Variance is reduced by an order of magnitude to 0.027. (b) For the optimal flow rate variance in absorption is reduced by an order of magnitude down to 0.0052. Note, that although metabolite outflux is penalized, it increased for all topologies to (a) 6.3%, (b) 1.9% and (c) 1.6%.
